# Fluvastatin Promotes Treg Cell Production in Allogeneic Immune Reaction and Suppresses Inflammatory Response

**DOI:** 10.1002/iid3.70165

**Published:** 2025-02-25

**Authors:** Xianxian Chen, Dong Huang, Li Zhao, Donghai Tang, Yu Tian, Chunxiao Ren, Fen Yan, Kailin Xu, Kai Zhao

**Affiliations:** ^1^ Department of Hematology The Affiliated Hospital of Xuzhou Medical University Xuzhou Jiangsu China; ^2^ Blood Diseases Institute, Xuzhou Medical University Xuzhou Jiangsu China; ^3^ Department of Orthopedics The Affiliated Hospital of Xuzhou Medical University Xuzhou Jiangsu China

**Keywords:** allogeneic hematopoietic stem cell transplantation, inflammation, statin, Treg cell

## Abstract

**Introduction:**

Statins, a class of HMG‐CoA reductase inhibitors, exhibit prophylactic benefits against immune rejection induced by allogeneic hematopoietic stem cell transplantation (allo‐HSCT). Despite the protective function is confirmed, the precise mechanism to induce immune tolerance of statin in the initial stages of transplantation remains incompletely understood. Given that Treg cells play a critical role in preventing graft versus host response and Foxp3 as a transcription factor of Treg can be induced by statins, we hypothesize that the immunosuppressive effects of statins are partially mediated through regulation of Treg cells expansion.

**Methods:**

T cells were stimulated in vitro under anti‐CD3/anti‐CD28/IL‐2/TGF‐β condition or allo‐reactive system with or without the addition of statins. The induction of Tregs were detected using flow cytometry. Allo‐HSCT models were established by transferring donor cells alone or combined with recipient treated by fluvastatin. The proportions of Treg and phenotypes of effector T cells were identified. Cytokine secretion and antigen‐presenting cell (APC) function were tested in irradiated mice.

**Results:**

Statins induced higher Treg production in classical and allogeneic cell co‐culture conditions in vitro. In the early stage of models treated with fluvastatin only in donors or combined treatment of donors and recipients, a similar phenomenon was observed with elevated levels of Foxp3^+^ Treg along with increased expression of CCR7, CD62L, and S1P1 on allo‐reactive T cells. Fluvastatin treatment suppressed the secretion of pro‐inflammatory cytokines IFN‐γ and TNF‐α by CD4^+^ and CD8^+^ T cells in irradiated mice. Furthermore, fluvastatin also contributed to restraining the numbers and activation of APCs, including dentritic cells (DCs) and macrophages in vitro and in vivo.

**Conclusion:**

Our finding demonstrated that statin exposure modulates immune responses during the initial phase of allo‐HSCT by promoting Treg expansion and suppressing inflammatory reactions, which supply a promising strategy for aGVHD prevention.

## Graphical abstract

Statins promoted Treg expansion both under steady‐state activation and allogeneic response conditions. Fluvastatin showed long‐lasting effects on upregulation of Treg cell number and reactive T cell phenotypes. IFN‐γ and TNF‐α secretion were inhibited by fluvastatin. Fluvastatin also contributed to restrain the numbers and activation of APCs including DCs and macrophages in vitro and in vivo.

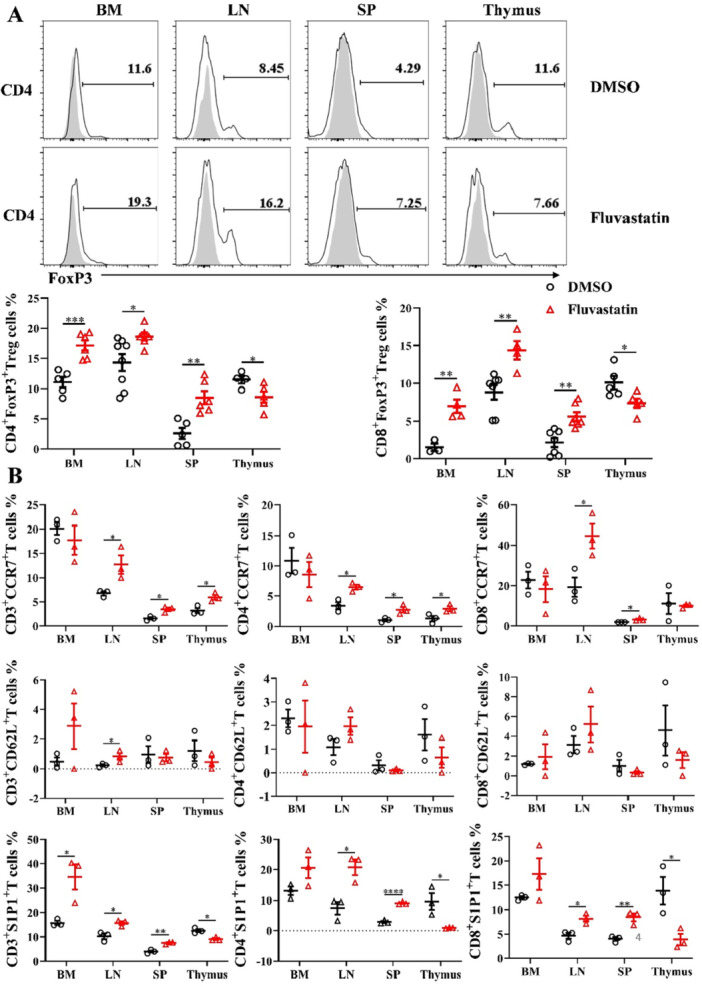

## 1 Introduction

Reaching and maintaining transplant tolerance is the ultimate objective following allogeneic hematopoietic stem cell transplantation (allo‐HSCT) and organ transplantation [1]. Statins as inhibitors of the activity of 3‐hydroxy‐3‐methyl‐glutaryl‐CoA (HMGCoA), exhibit potent immunomodulatory and anti‐inflammatory properties [2]. Extensive preclinical and retrospective clinical data suggest that statins have the potential to prevent acute graft versus host disease (aGVHD) while preserving graft versus leukemia (GVL) effects [3, 4, 5]. Increasing evidence supports the use of statins as a prophylaxis for GVHD after allo‐HSCT [6, 7, 8]. Mechanically, statins show their ability to prevent aGVHD through multiple mechanisms including: (i) modulating the balance of CD4^+^ Th cell subsets [9], (ii) inducing downregulation of co‐stimulatory molecules and MHC Ⅱ expression on APCs [10], (iii) preventing donor T cells from homing to lymph nodes and peyer's patches [11, 12]. Statins hold promise in influencing the development of aGVHD from various perspectives [13]. However, the determination of the potential impact of statins on inducing immune tolerance to attenuate transplant rejection during the initial stage of allo‐HSCT remains to be elucidated.

Regulatory T cells (Treg) characterized by Foxp3 expression devoted to maintaining immune tolerance and inhibiting GVHD [14, 15, 16]. Due to the limited availability of peripheral Treg cells, considerable efforts have been directed towards expanding Treg cells either in vivo or ex vivo for therapeutic applications [17, 18, 19, 20]. Our utilization of engineered Treg cells for the prevention of murine GVHD presents challenges due to the complex manufacturing process, which may limit their application in allo‐HSCT patients [21]. However, the encouraging finding demonstrated the potential of statins to induce and recruit Treg cells in vivo [22, 23]. In vitro experiments have also shown that statins significantly impact various aspects of Treg biology and the phenotype of conventional T cell [24]. Therefore, we hypothesize that the protective effect of statins on allo‐HSCT may partially attribute to the regulation of Treg cell and effector T cell division.

Treg cell not only modulates the magnitude of immune response, contributing to immune tolerance, but also restrains inflammatory response by modulating the activity of a wide range of adaptive and innate immune system components [25]. The cytokine storm, which is a side‐effect of pre‐conditioning irradiation, serves as the initial trigger for aGVHD by activating APCs [26, 27, 28]. Other studies have reported that statins can attenuate the inflammatory functions and pathogenic activity of T cells [29]. In our allo‐HSCT model, we observed a reduction in effector T cell secretion of inflammatory cytokines in recipients after transplantation with donor pre‐treated with fluvastatin [12]. Additionally, it has been demonstrated that statins have the capacity to inhibit APC function in both murine models and human [9, 10]. Therefore, we predicted that statins could potentially prevent irradiation‐induced cytokine secretion or would suppress APC activation.

Based on these concerns, the effects of stains on the induction of Treg cells was assessed in an allogeneic reactive cell co‐culture system and an allo‐HSCT murine model, while also characterizing effector T cell properties. We illustrated here that statins significantly augment the population of Treg both during homeostatic activation and in response to allogeneic stimuli. Additionally, they concurrently suppress cytokine secretion and APC activation induced by irradiation. Our findings further elucidate the potential mechanisms underlying the effects of statins, highlighting their ability to augement Treg and suppress inflammatory responses as promising strategies for mitigating aGVHD.

## 2 Materials and Methods

### 2.1 Mice

The female C57BL/6J (H‐2K^b^, 8–10 weeks) and BALB/c (H‐2K^d^, 8–10 weeks) mice were purchased from Vital River Laboratory Animal CO. Ltd (Beijing, China). The mice were housed in a Specific Pathogen Free facility at Xuzhou Medical University under controlled conditions of constant temperature (25 ± 2°C) and relative humidity (55%). All animal experiments were approved by the Medical ethics committee of the Xuzhou Medical University (IACUC Issue No. 202208S094), and according to the ARRIVE guideline.

### 2.2 Reagents

Fluvastatin was purchased from Dalian Meilun Company, and Simvastatin was obtained from MCE Company. Mouse anti‐CD3 (145‐2C11), ‐CD4 (RM4‐5), ‐CD8 (53‐6.7), ‐CD25 (3C7), ‐FoxP3 (MF‐14), ‐MHC II (M5/114.15.2), ‐CD11c (N418), ‐F4/80 (BM8), ‐CCR7 (4B12), ‐CD62L (MEL‐14), ‐IFN‐γ (XMG1.2), ‐TNF‐α (MP6‐XT22) antibodies were purchased from Biolegend. The mouse anti‐S1P1 (713412) was acquired from R&D Company, while the anti‐CD80 (16‐10A1) antibody was purchased from eBioscience. Anti‐CD3 mAb and anti‐CD28 mAb were obtained from BioGems. The cytokine IL‐2 was purchased from Biolegend, and TGF‐β was purchased from MCE.

### 2.3 Cell Sorting

Bone marrow cells from femur and tibia were prepared and T cells were depleted using CD90.2 Positive Selection Kit (Stemcell Technologies, Shanghai, China) according to the manufacturer's instructions. Splenic and lymph node T cells were isolated and purified using the EasySep negative selection Kit (StemCell Technologies). The purity of CD3^+^ T Cell exceeded 95%.

### 2.4 Ex Vivo Induction of Tregs

Purified T cells were pre‐incubated with or without Simvastatin (2 μmol/L) for 12–18 h at 37°C, and subsequently activated using plate‐bound anti‐CD3 antibody (2 μg/mL) and soluble anti‐CD28 antibody (2 μg/mL), or left un‐activated. Following activation, T cells were cultured in complete medium containing IL‐2 (5 ng/mL), in the presence or absence of TGF‐β (5 ng/mL) for 2‐5 d to induce differentiation. The total T cells were harvested and stained with anti‐CD3, anti‐CD4/CD8, anti‐CD25, and anti‐Foxp3 antibodies to identify Foxp3 positive cells.

### 2.5 T Cells and APCs Co‐culture in Vitro

Isolated CD3^+^ T cells from C57BL/6J mice using EasySep negative selection reagents and splenic antigen presenting cells (APCs,) from BALB/c mice were isolated following T cell depletion using the CD90.2 Positive Selection Kit. The CD3^+^ T cells and APCs were co‐cultured at a concentration of 4 × 10^6^ cells/mL at 37°C in the presence or absence of Fluvastatin (2 μmol/L) or Simvastatin (2 μmol/L), respectively, in a complete medium containing IL‐2 (5 ng/mL) for 24 and 48 h. Subsequently, the incubated cells were stained for flow cytometry detection.

### 2.6 Allogeneic Transplant Model

C57BL/6J mice were utilized as donors and pre‐treated with intraperitoneal injections of Fluvastatin (20 mg/kg, 200 μL/mouse) or DMSO by once daily for 7 consecutive days. BALB/c recipients underwent total body irradiation (TBI) with 7.5 Gy of ^60^Coγ radiation, followed by the administration of donor‐derived BM cells (6 × 10^6^ cells/mouse) and purified T cells (2 × 10^6^ cells/mouse) to induce allogeneic immune responses. The transplantation was performed on day 0, and the recipients were administered either Fluvastatin (40 mg/kg/mouse/day) in their drinking water or not. On the 4th and 6th days post‐transplantation, the Foxp3‐labeled Treg cells and surface receptors of effector T cells in the BM, LN, spleen, and thymus of the recipient mice were detected.

### 2.7 Irradiation Model

BALB/c mice were intraperitoneally administered Fluvastatin or DMSO injection (20 mg/kg, 200 μL/mouse) once a day for 3 consecutive days in a randomized manner. Subsequently, total body irradiation (TBI) with 3.5 Gy of ^60^Coγ was given twice a day. The initiation of irradiation was designated as day 0, and the mice were killed on the 1st and 3rd days post‐irradiation. Flow cytometry was employed to assess cytokine production by T cells and activation of DCs in BM, LN, and spleen.

### 2.8 Flow Cytometry

Single‐cell suspensions were harvested from BM, LN, and spleen. The cells were stained for viability assessment and cell surface markers. For intracellular cytokines staining, the cells were stimulated with PMA (50 ng/mL, Sigma) and Ionomycine (750 ng/mL, Sigma) in the presence of Brefeldin A (10 μg/mL, Invitrogen) at 37°C for 4 h. Flow cytometry analysis was performed using a BD LSR Fortessa Flow Cytometer (RRID:SCR_019601). Data were analyzed using FlowJo software (FlowJo X, BD Inc. RRID:SCR_008520).

### 2.9 QRT‐PCR

According to the instructions of Ambion's Trizol to extract total RNA from splenic cells. Subsequently, the obtained total RNA was reverse‐transcribed into cDNA using the M‐MLV reverse transcriptase kit (Invitrogen) following standard procedures. RT‐PCR analysis was performed on Roche Applied LightCycler 480 (Roche) with SYBR Green Master Mix (Vazyme), employing GAPDH as an internal reference in each sample. The following primer sets were used: KLF2 F 5′‐ACA GAC TGC TAT TTA TTG GAC CTT AG‐3′, R 5′‐CAG AAC TGG TGG CAG AGT CAT TT‐3′; GAPDH F 5′‐TTG ATG GCA ACA ATC TCC AC‐3′, R 5′‐CGT CCC GTA GAC AAA ATG GT‐3′.

### 2.10 Statistical Analysis

Statistical analyses and graphs were performed using GraphPad Prism 8 software (GraphPad Prism, SanDiego). The data were presented as the mean ± standard deviation without any exclusions. The comparison between two groups was performed using the Student *t*‐test or non‐parametric test, while One‐way ANOVA was employed for multiple group comparisons. Value of *p* < 0.05 was considered statistical significance.

## 3 Results

### 3.1 Simvastatin Increased Foxp3^+^ T Cell Production In Vitro

**Figure 1:**
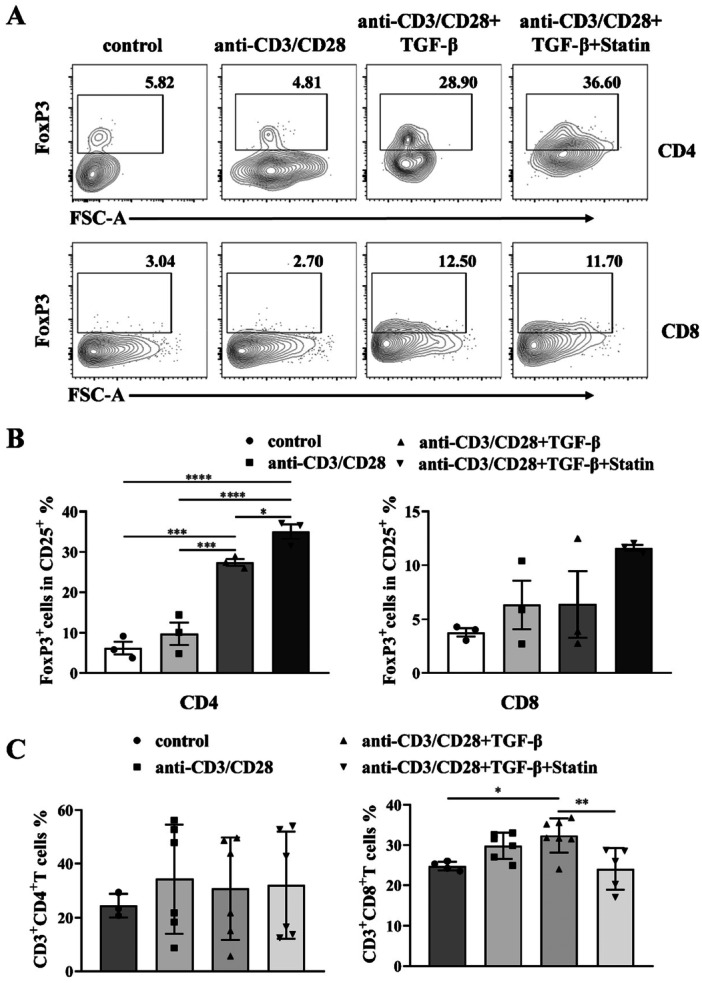
Simvastatin induced Treg production in vitro. (A) The representative flow data of FoxP3^+^ cells in CD25 positive CD4^+^ and CD8^+^ T cells were shown. (B) Expression of FoxP3 in CD25 positive CD4^+^ and CD8^+^ T cells. (C) Percentages of CD4^+^ and CD8^+^ T cell after 2–5 days’ incubation in vitro; Three independent experiments were repeated. Each symbol presents the data from one well. * *p* < 0.05, **p < 0.01, ***p < 0.001, ****p < 0.0001.

To investigate the impact of statin on the induction of immune suppressive T cells, we evaluated the percentage of Foxp3^+^ T cells in naïve T cell stimulated with anti‐CD3/anti‐CD28/IL‐2/TGF‐β in the presence or absence of simvastatin. As depicted in Figure 1A, both CD4^+^ and CD8^+^ T cells exhibited increased levels of Foxp3 when treated with statin compared to those without Simvastatin. However, Foxp3^+^ cells were increased more significantly in CD25^+^CD4^+^ T cells (Figure 1B). To account for potential variations in cellular composition induced by statin, proportions of CD3^+^ T subsets were detected. No significant differences were observed in the percentages of CD4^+^ T cells and lower CD8^+^ T cells in stimulus with statin (Figure 1C). These findings demonstrated that Simvastatin could induce the generation of Treg cells in vitro, independent from the alterations in CD4^+^ or CD8^+^ T cell subpopulations.

### 3.2 Upregulated Tregs Expansion and Decreased Allo‐Reactive Activity Were Induced by Statins in Allogeneic System

**Figure 2:**
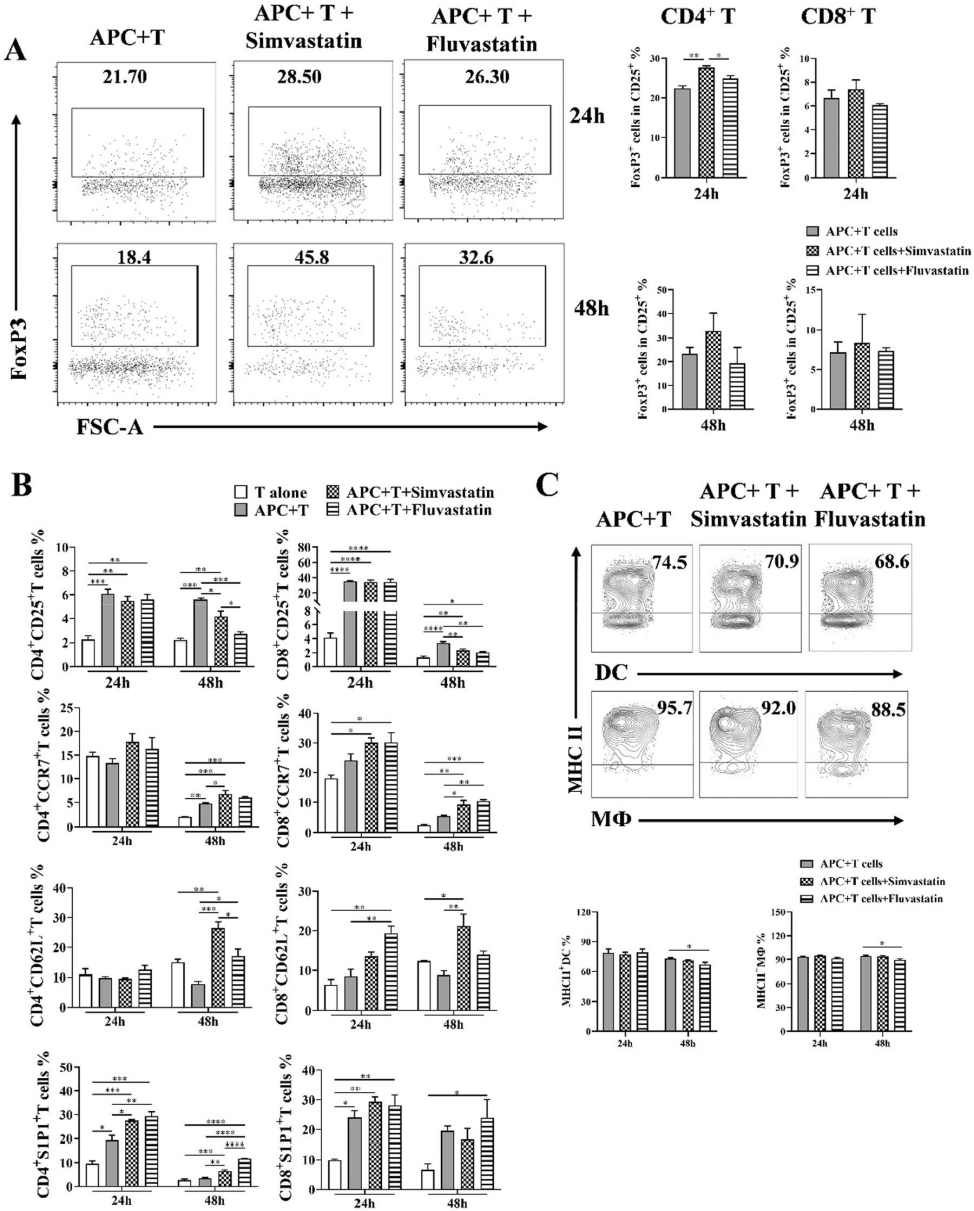
Statins regulated allo‐reactive T and APC responses in allogeneic cell co‐culture systems. (A) The representative dot plots and statistical analyzed data of FoxP3^+^ cells in CD25^+^ CD4^+^ and CD8^+^ T cells after 24 and 48 h culture. (B) Expression of T cell surface markers in stains present or absent incubation in vitro. (C) MHC II levels on DC and macrophage after 48 h co‐culture with allogeneic T cells with or without statins. Two repeated experiments were done. *p < 0.05, **p < 0.01.

Subsequently, we conducted an in vitro investigation to elucidate the impact of statins on allogeneic responses. The ratio of CD4^+^ to CD8^+^ T within the CD3^+^ T cells remained unchanged following statin treatment compared to the absence of statin (Figure S1A). In the presence of simvastatin, there was a significant increase in the percentages of Foxp3 expression on CD25^+^CD4^+^ T cells compared to the APC + T and T cell alone groups. Similarly, Fluvastatin also exhibited a similar elevated trends as Simvastatin, leading to an enhanced generation of Foxp3^+^ Treg cells (Figure 2A). Then we aimed to reveal the characteristics of allo‐reactive T cell following statin treatment. As shown in Figure 2B, the proportion of CD25^+^ T cells remarkably increased upon co‐culture with APCs. Compared with APC + T group, the CD25^+^ T proportion was significantly decreased at 48 h but not at 24 h. Both simvastatin and Fluvastatin significantly enhanced the expression of the homing receptor CCR7 on CD4^+^ and CD8^+^ T cells after 48 h, while no significant difference was observed at 24 h. The addition of statins resulted in elevated levels of CD62L on both CD4^+^ and CD8^+^ T cells (Figure 2B). The expression of S1P1, responsible for T cell egress from lymph nodes and thymus, were detected [30]. The surface expression of S1p1 on CD4^+^ T cells was significantly upregulated by statins, as shown in Figure 2B, after both 24 and 48 h of culture. However, no significant differences were observed in the levels of S1P1 on CD8^+^ T cells between the groups with and without statins at these two time points. Furthermore, the expression of MHC Ⅱ on CD11c^+^ DCs and F4/80^+^ macrophages was detected, respectively. Reduced levels were observed in APC + T cell with Fluvastatin treatment at 48 h, both in DCs and macrophages, compared to cells without Fluvastatin. However, no changes were detected at 24 h (Figure 2C). These data suggested that statin treatment enhances allogenic immune suppression.

### 3.3 Fluvastatin Promoted Treg Cells Generation in Allo‐HSCT Recipients

**Figure 3:**
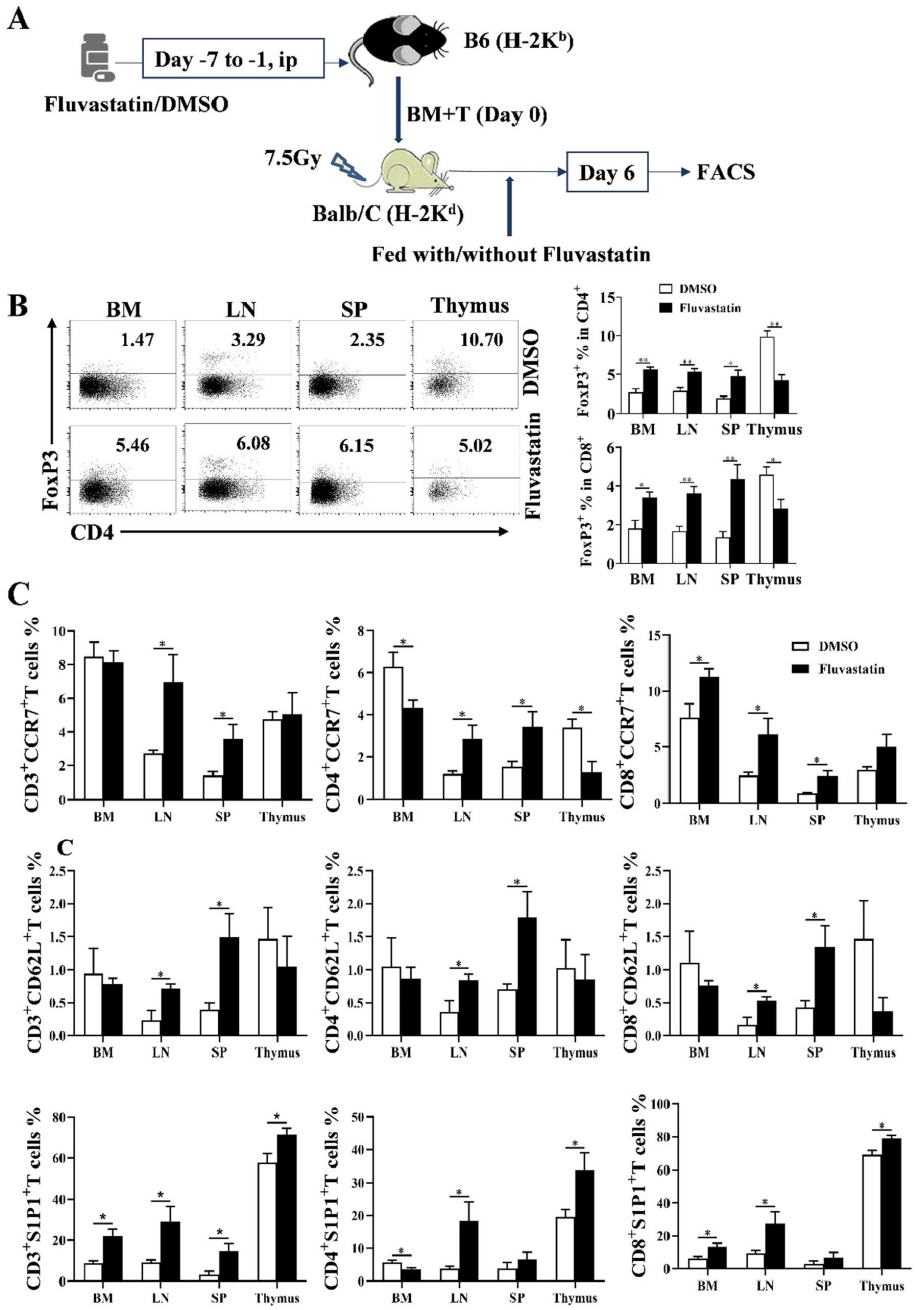
Fluvastatin increased the percentages of Treg and regulated effector T cell homing receptors. (A) Experiment design of allo‐HSCT model. Donors were pre‐treated with fluvastatin, and recipients were fed with or without Fluvastatin were used. (B) Percentages of Treg cells in BM, LN, SP, and thymus after transplantation for 6 days. (C) T cell migration related receptors were detected by flow cytometry. The percentages of CCR7, CD62L, and S1P1 positive CD4^+^ and CD8^+^ T cells were calculated. Experiments were repeated twice and each group had three mice. *p < 0.05.

Although statins promote the expansion of Treg from allo‐reactive cultures in vitro, it remains unclear whether the in vivo requirements for stains and Ag‐driven expansion of Treg differ. To determine the levels of Treg cells in an allo‐HSCT model, C57BL/6J mice were used as donors with or without pre‐injection fluvastatin. Following transplantation, recipients were continuously administered fluvastatin or not (Figure 3A). The administration of fluvastatin resulted in two‐three folds’ increase in the expression of Foxp3 in CD4^+^ T cells from BM, LN, and SP compared to control mice. However, a lower percentage of Foxp3 was observed in the thymus. Furthermore, a similar trend was investigated in Foxp3^+^CD8^+^ T cells (Figure 3B). We subsequently conducted a comparative analysis of allo‐reactive T cells phenotypes, with a particular focus on receptors associated with the migration. As depicted in Figure 3C, both CD4^+^ and CD8^+^ T cells exhibited significantly higher expression of homing receptors CCR7 and CD62L in LN and SP compared to the control groups. However, no alterations were observed in BM and thymus, which serve as central immune organs. Notably, luvastatin treatment led to widespread upregulation of S1P1, potentially facilitating the egress of Treg from the thymus while promoting retention of allo‐reactive T cell in SLOs.

### 3.4 Fluvastatin Had Long‐Term Effects on Remolding T Cell Subset and Characteristics

**Figure 4:**
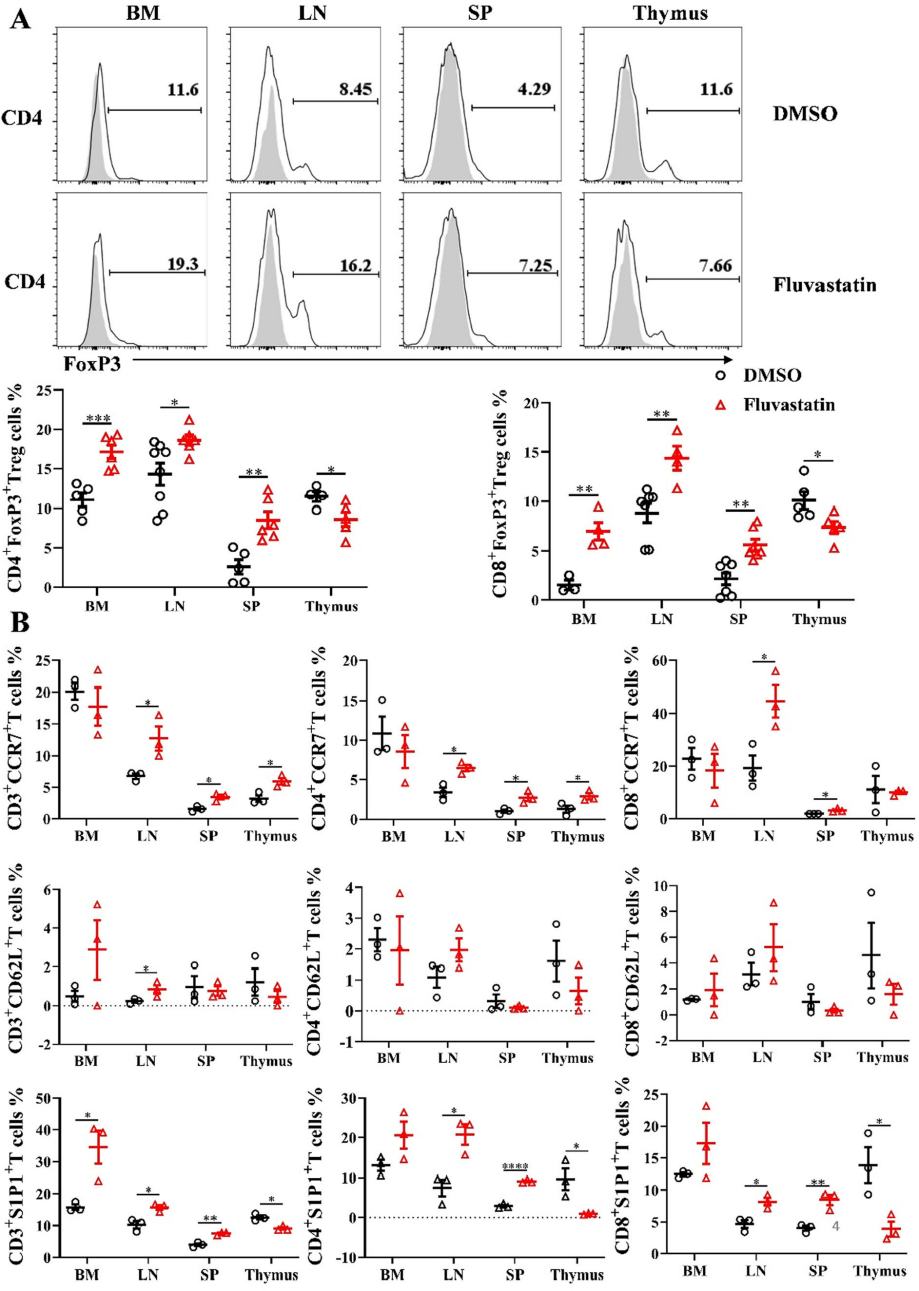
Donors exposure to Fluvastatin conferred preferential upregulation of Treg cell number in transplanted recipients. Donors were pre‐treated for 7 consecutive days, and then BM and T cells were harvested to do transplantation. Treg cells and surface markers were detected on day 4 after transplant by flow cytometry. (A) Percentages of CD4^+^ and CD8^+^ Treg cells in BM, LN, spleen and thymus. (B) CCR7, CD62L, and S1P1 positive CD4^+^ and CD8^+^ T cells were detected. Two independent experiments were repeated and each symbol presented one mouse. Experiments were repeated fifth and each group had 3 mice. *p < 0.05, **p < 0.01, ***p < 0.001, ****p < 0.001.

To ascertain the transient or enduring effects of Fluvastatin on T cells, donors were subjected to pre‐treatment before transplantation while the recipients did not receive any administration of Fluvastatin. Consistent with the previous data, representative data demonstrated that an increase in foxp3 expression in mice pre‐treated with Fluvastatin (Figure 4A). Notably, higher percentages of CD4^+^ Treg and CD8^+^ Treg in BM, LN, and SP were observed. As expected, Fluvastatin administration led to a reduction of CD4^+^ Treg and CD8^+^ Treg in thymus probably due to their elevated output to peripheral lymphoid organs. Similarly, CCR7 expression on CD4^+^ and CD8^+^ T cells was increased in LN and SP compared to control mice. However, there was no statistically significant difference in the level of CD62L with or without Fluvastatin treatment (Figure 4B). S1P1 exhibited higher expression in LN and SP, however, contrary to previous data, lower levels were found in thymic CD4^+^ and CD8^+^ T cells (Figure 4B). These findings indicated that although only the donors received pre‐transplantation Fluvastatin, enhanced induction and maintenance of Treg were mediated by Fluvastatin.

### 3.5 Effects of Statins on Irradiation Induced Immune Activation Model

**Figure 5:**
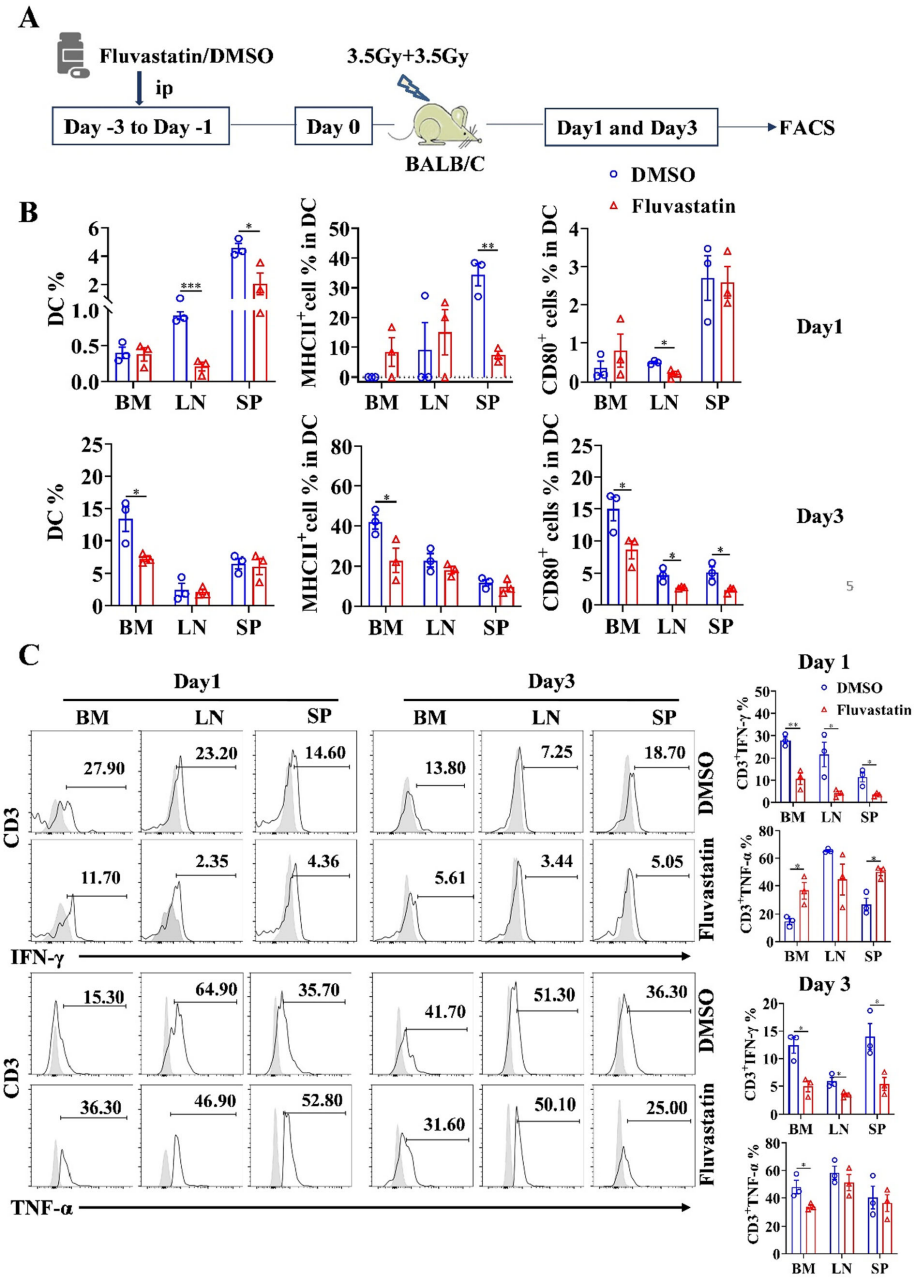
Statins decreased the cytokines production and the activity of APCs in the irradiated mice. (A) Experiment design was shown. (B) Percentages and activation of DC in BM, LN, and spleen of irradiated mice were evaluated. (C) Cytokine secretion of T cells were detected on days 1 and 3, respectively. The representative flow histograms and statistical data were shown. *p < 0.05, **p < 0.01, ***p < 0.001.

TBI is a commonly employed pre‐conditioning regimen before allo‐HSCT, which triggers sterile damage associated molecular patterns (DAMPs) driving innate and adaptive immune responses [31, 32]. The upregulation of MHC and co‐stimulatory molecules on myeloid cells, in conjunction with the release of pro‐inflammatory cytokines, serves as the initiation mechanism for GVHD during the early stages following allo‐HSCT [33, 34]. We subsequently evaluated the suppressive capacity of Fluvastatin in a myeloablative irradiation model. For that purpose, BALB/c mice were pre‐treated with Fluvastatin for 3 days before receiving a dose of 7 Gy irradiation (Figure 5A). Following irradiation for 1 day, a reduction in DC population was observed in both LN and SP of Fluvastatin‐treated mice, accompanied by suppressed levels of MHC II expression on SP‐derived DCs and decreased CD80 expression on LN‐derived DCs (Figure 5B). Three days later, a decrease in DC levels was evident within the BM, along with a notable downregulation of MHC II expression, particularly CD80 (Figure 5B). The production of IFN‐γ and TNF‐α by CD3^+^ T cells were assessed using flow cytometry on days 1 and 3 post‐irradiation. Representative data in Figure 4C demonstrated that Fluvastatin treatment reduced the expression of IFN‐γ in BM, LN, and SP on days 1 and 3. Secretion of TNF‐α in CD3^+^ T cells from BM and SP was upregulated on day 1, while a lower level was observed in BM on day 3 compared to control groups (Figure 5C). There was no significant alteration observed in the percentage of CD4^+^ and CD8^+^ T cells (Figure S2). The aforementioned data suggests that the immunosuppressive effect of statins holds potential benefits in safeguarding the host against cytokine release syndrome and severe GVHD.

## 4 Discussion

Our and others’ research has demonstrated the potential of statins in preventing or alleviating transplant rejection in both murine and human studies [12, 35, 36]. However, further investigation is warranted to elucidate the mechanism of immune tolerance, considering the diverse immunomodulatory functions exerted by statins. The functional suppression of allo‐reactive lymphocytes by Treg is a crucial mechanism for maintaining immune tolerance. To effectively inhibit the expansion of allo‐reactive T cells and prevent GVHD occurrence, early‐stage production of Treg is indispensable in the context of allo‐HSCT. In the present study, we found that Fluvastatin treatment induced a significant increase in CD4^+^ and CD8^+^ Treg 4–6 days after transplantation. Eric et al reported that KLF2 was necessary for the generation of antigen‐induced Tregs (iTreg) generated in SLOs to promote FoxP3 transcription, and their in vivo counterpart, peripheral Tregs (pTreg) [37]. Consistent to our previous data [12], we confirmed that Fluvastatin could obviously upregulated the levels of KLF2 (Figure S3). Although the precise underlying mechanism through which statins promote Treg cell production was not investigated in this study, these aforementioned data may elucidate the upstream mechanism of stain‐induced Treg production. Moreover, it was demonstrated that KLF2 is specifically essential for the generation of iTreg rather than thymus‐derived Tregs, thereby effectively illustrating the observed phenomenon of Treg proportions increased in BM, LN and spleen, while decreased in thymus.

Statin pre‐treatment of both the donor and recipient was found to enhance the protective effect against aGVHD‐associated mortality, which was used as potentially effective strategy of prophylaxis [9, 38]. However, it has been reported that only statin treatment in the donor is sufficient to prevent severe aGVHD, and the protective effect of statin on GVHD is limited to recipients [5]. To further explore the potential rationale, we designed allo‐HSCT models in which donors were pre‐treated with Fluvastatin, with or without continuous administration of statins to recipients. The results presented here demonstrated that regardless of whether it was administrated to recipients or not, exposure of the donor to Fluvastatin can lead to increased production of Treg. Moreover, we observed upregulation of CCR7, CD62L and S1P1in both recipient groups, indicating a potential dependence on higher expression levels of KLF2 [39, 40]. In other words, the immunological effects of statins on donor‐derived T cells can be maintained after adoptive transplantation into recipients, resulting in an increased number of Treg cells and phenotypic modifications over the long term.

Pre‐conditioning with irradiation leads to an inflammatory environment within the BM, thereby initiate allogeneic immune rejection and exerting detrimental effects on the HSC engraftment. In the current irradiation model, Fluvastatin was found to suppress DC number and activation, which were coincided with the reduced secretion of IFN‐γ and TNF‐α during the initial inflammatory process following conditioning. The suppressive effects on APC function are likely to contribute to subsequent prevention of immune rejection. Additionally, previous data have demonstrated that Simvastatin can serve as an effective agent targeting the bone marrow niche, enhancing HSC engraftment and expansion of the donor stem cells [41]. Collectively, these data provide compelling evidence supporting statins as potential therapeutic agent for alleviating inflammatory BM conditions, promoting immune tolerance, and improving engraftment.

In summary, these data demonstrate that statin treatment significantly enhances Treg production both in vitro and in vivo, exhibiting sustained functionality exclusively under donor pre‐treated conditions. Moreover, efficient suppression of pro‐inflammatory cytokine and APC activation also contributes to preventing graft versus host response. However, the present study does not elucidate the relationship between Treg and the reduced production of pro‐inflammatory cytokine following statin treatment, which warrants further exploration.

## Author Contributions

**Xianxian Chen:** data curation, formal analysis, and methodology. **Dong Huang:** data curation, formal analysis, and methodology. **Li Zhao:** data curation, formal analysis, and methodology. **Donghai Tang:** methodology and validation. **Yu Tian:** Methodology. **Chunxiao Ren:** methodology and validation. **Fen Yan:** methodology and validation. **Kailin Xu:** supervision and writing–review and editing. **Kai Zhao:** conceptualization, project administration, writing–original draft, and writing–review and editing.

## Ethics Statement

Animal experiment was approved by the Medical ethics committee of the Xuzhou Medical University (IACUC Issue No. 202208S094), Xuzhou, Jiangsu, China. Best efforts were undertaken to minimize animal suffering. All the experimental procedures used in this study were carried out according to the experimental animal protocol approved by the ethics committee.

## Conflicts of Interest

The authors declare no conflicts of interest.

## Supporting information

Supporting information.

Supporting information.

Supporting information.

Supporting information.

## Data Availability

The authors have nothing to report.
