# Supplementary figures and images for: Fluvastatin Promotes Treg Cell Production in Allogeneic Immune Reaction and Suppresses Inflammatory Response
Source: Immun Inflamm Dis. 2025 Feb 25;13(2):e70165. doi: 10.1002/iid3.70165 (PMC11861029; doi:10.1002/iid3.70165)

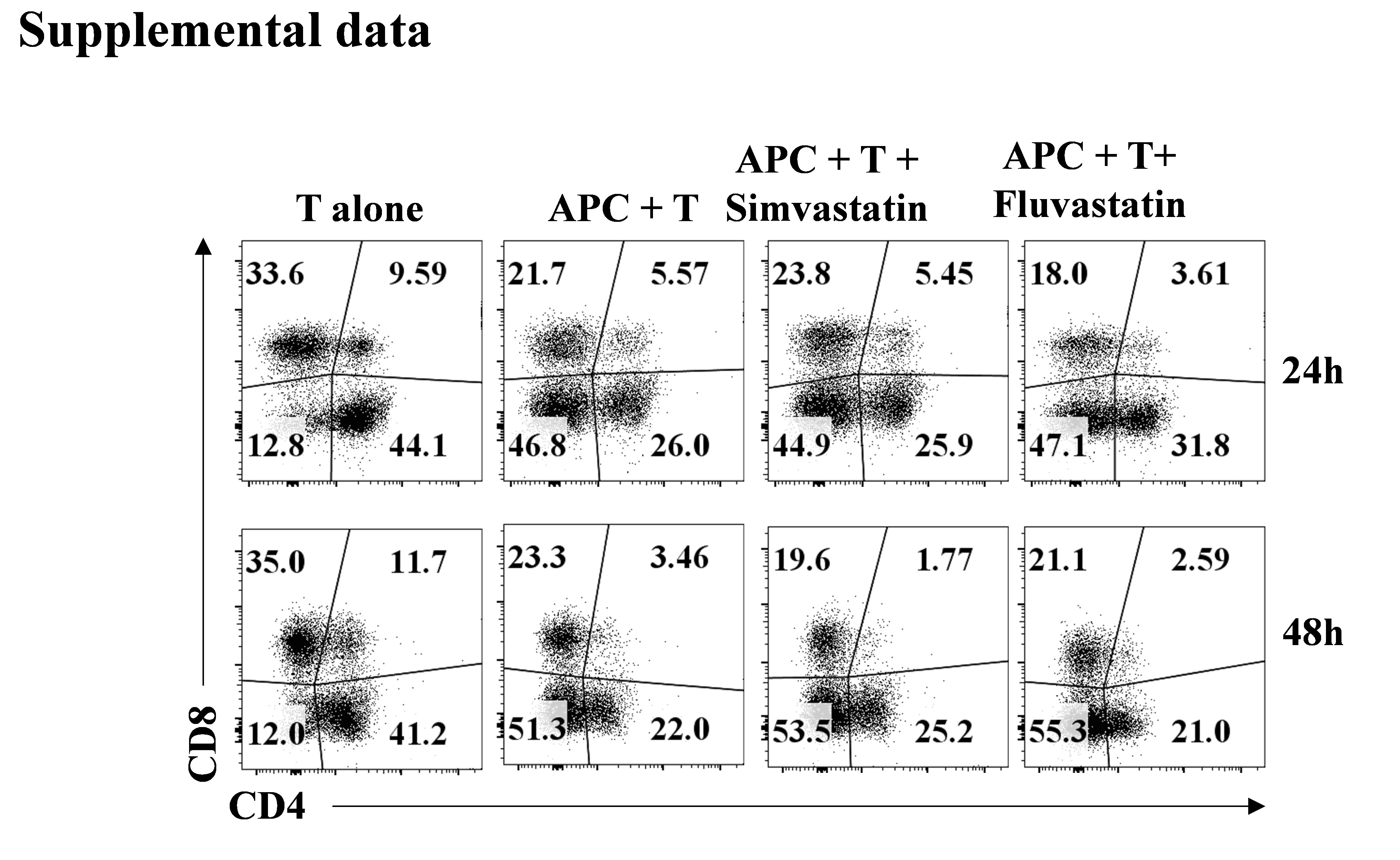

Supplement: Supplementary file 1 — Supporting information. [file IID3-13-e70165-s004.jpg]

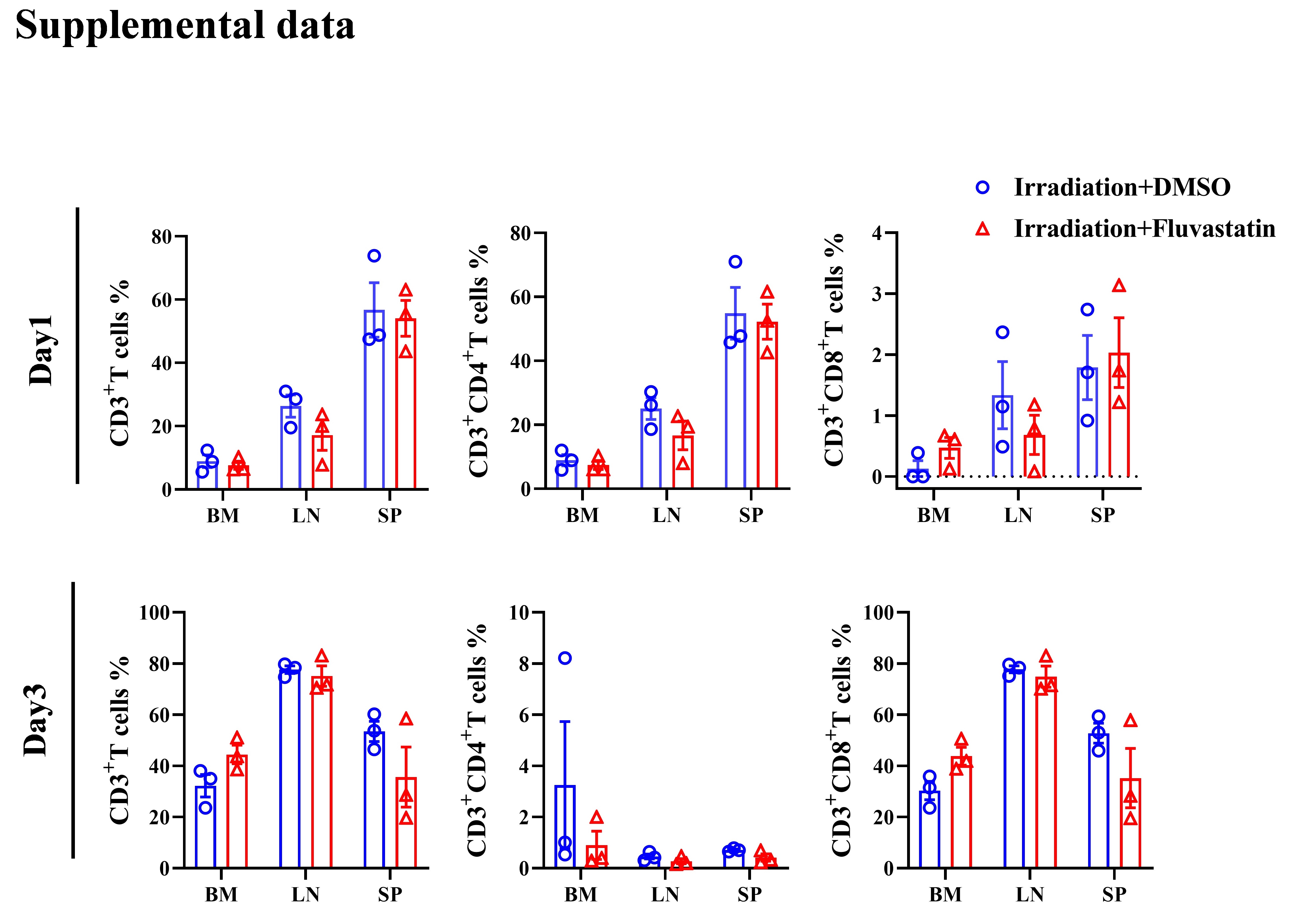

Supplement: Supplementary file 2 — Supporting information. [file IID3-13-e70165-s001.jpg]

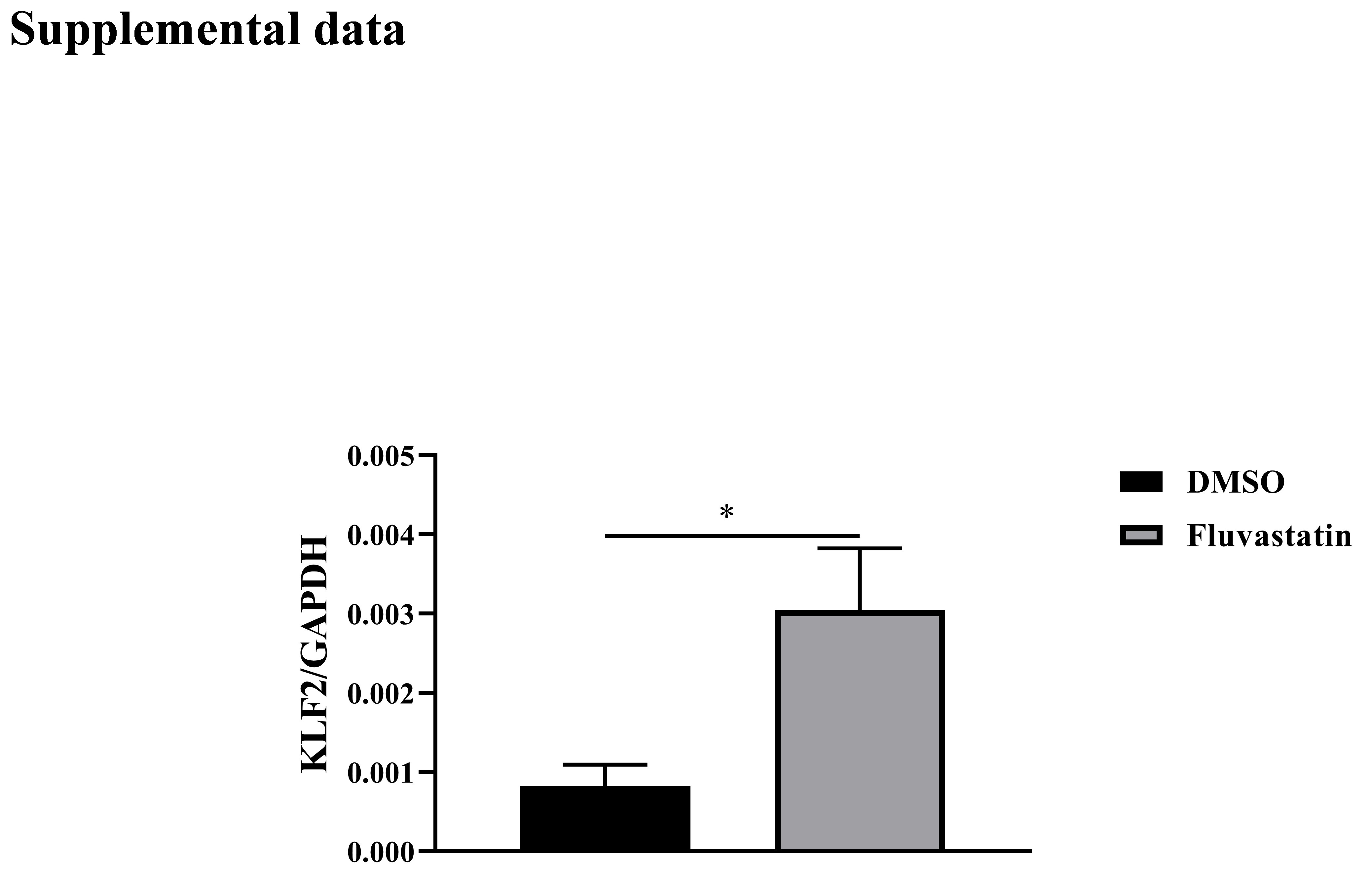

Supplement: Supplementary file 3 — Supporting information. [file IID3-13-e70165-s003.jpg]

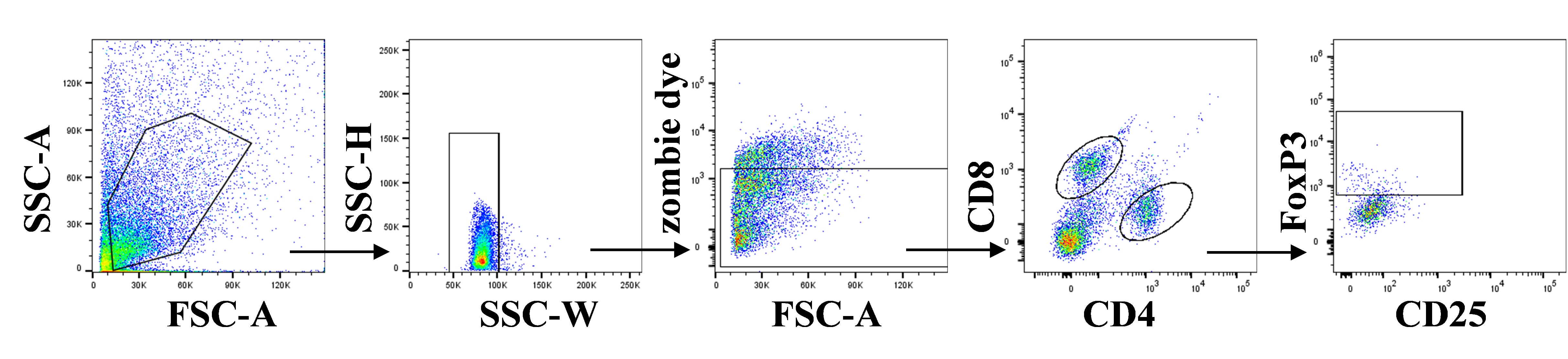

Supplement: Supplementary file 4 — Supporting information. [file IID3-13-e70165-s002.jpg]
